# The Doctor, the Public and Medical Legislation*Read Before the Georgia State Medical Association at Athens.

**Published:** 1910-06

**Authors:** J. G. Dean

**Affiliations:** Dawson, Ga.


### THE DOCTOR, THE PUBLIC AND MEDICAL LEGISLATION.*

**Read Before the Georgia State Medical Association at Athens.*

BY DR. J. G. DEAN, DAWSON, GA.

Regarding much that is written and said on such occasions as this as bearing many of the ear-marks of plagiarism or repetition of things said or written about many times before, it has been usually my habit to observe silence, being content to listen to others. There are, however, many things which never grow old, and which should not bore though often repeated. I am sure that I shall say things here which are familiar to all doctors. For this I offer no apology. There are some things about which even doctors should be reminded. There are certain conditions abroad in Georgia which it seems to me appeal to every thinker on medical and especially prophylactic lines. These conditions concern not only doctors but the laity, as well, and demand, as I see the matter, important legislation both general and local, hence the rather broad subject selected by me for this occasion. Since the days of Aesculapius, Hippocrates, Pythagoras, and Galen the doctor and his art have been important factors among the public and in matters of legislation. As the years have passed observation and experience have taught mankind much as to disease and its remedies. The world has now come more than ever to rely on the wisdom of the physician, hence the added reason why he, the physician, should measure up to constantly growing expectations. Advance in public education and culture brings to the physician increased responsibility. The day is approaching, and rapidly, when, by reason of the better education of the public the isms and pathies in medicines which have played so great a part in the past will find deception more difficult. The public can ever be duped for a time, some of it all the time, but education brings reason, begets selection, which selection falls upon the most meritorious. The physician who has not in entering upon what is one of the most erudite of the professions, and in which there are the greatest of all responsibilities, taken the pains and the time to properly fit himself in literary attainment, has failed utterly to properly estimate his supposed aim in life and richly deserves the frequent failures that come the way of so many followers of the Aesculapian art. Every physician should be second in education and culture to no man in his community, he should strive to constitute himself the criterion educationally of his surroundings. There are many things which indicate necessity for change in medical requirement and in medical legislation. No one recognizes this so soon as the thinking physician, but where should the change begin? Where should we go for the root of certain troubles? Thus far the public has proven rather indifferent to strictly medical legislation. Let there come disease which endangers the safety of the public-pocket-book through the loss of horses, cows, sheep or swine, and every farmer, merchant or professional man is a unit on any measure looking to the eradication of such pestilence regardless of cost. State legislatures and the National Congress are bombarded with appeals, if there is need, for some new enactment to aid in saving the dumb brutes, thus preventing pecuniary loss to their owners. We hear much about efforts being made at a cost to the general government of many thousands of dollars to eradicate the boll-weevil, to banish black root from cotton. All such things if allowed to spread mean pecuniary loss. But how many of the general public have shown an interest in preventing the spread of certain diseases now known to be amenable to control when proper restrictions are brought to bear by proper legislation, general, as well as local? How can the medical profession best succeed in eventually being able at all times to easily enlist the sympathy and co-operation of the public in efforts at disease prevention or restriction? My answer is in two words, "By Education." Let the medical profession direct much of its energies toward furthering general education and consequent public culture. Lt's look to the future and not be content with the ideals of the past or present. Let's aid in taking at all times such steps as mean not only advancement constantly in public knowledge, but professional attainment as well. Let's go to the seats of medical learning and demand that more attention be given to educational requirements. Why is there in America more than double the number physicians
there in America more than double the number of physicians proportion to population in America when compared to the same situation in leading countries abroad? In foreign countries something like the following in that regard is the situation: In Austria there is one physician to 2,500 people; in Germany one for every 3,000; Great Britain one for every 1652; France one for every 1814; Italy one for every 3,500; and in Canada, just across our border, one for every 1193 inhabitants. What about the situation in our own great America with its boasted civilization and wealth? There is one physician to every 600 of our population, and druggists in proportion! Think of the disparity! Why this state of affairs? There is a pertinent reason. That reason is vital, and reflects first on medical institutions of learning. These institutions should be made to mend their ways as to the literary requirements which they claim to impose on the proposed student who knocks at their door.

As far back as 1766 the Medical Society of New Jersey demanded that no student of medicine should be lacking in a good knowledge of Latin, and, at least an initiatory knowledge of Greek. This was in the right line, but do the facts of the present day indicate that our medical institutions are, when we are advancing along all other lines, adhering strictly to such educational requirements. A diploma in medicine is now supposed to be obtained after four terms in a medical college. That *should* mean much. Not long since I was called by telephone by a brother practitioner and the following question was asked: "Have you got *airy* instrument you use in"—giving the name of a certain operation requiring considerable care and technical skill in its performance, and stating that he had a patient needing this operation and he would send for the instrument." He had but recently completed four courses in two medical colleges and obtained his diploma. Another physician in attempting to tell of a patient he had which had been in a comatose condition for some hours, referred to the condition as a "*catamose*" one. Still another had made an insurance examination which certain circumstances caused me to have to scan and an absence of a knowledge of orthography or the rules which govern the use of capital letters was at once manifest. All these are graduates of reputable medical colleges! Then why wonder that there is great crowding of the medical profession in this country and frequent lack of proper regard for ethics among members of the profession? *Get after the colleges,* force them to improve their product, decrease the output by more stringent educational requirements and we will not only compare favorably in every respect, medical and surgical, with the older countries, but the public will entertain greater respect for the medical man, our influence for good will be greater and we can more easily produce results when medical legislative enactments are wanted. The medical profession of Georgia is at this time earnestly engaged in a warfare on the fly, the hookworm, the mosquito, and other enemies to the health of mankind. To win signal victory in this very laudable medical effort we must have the co-operation of the public that we may be able to secure medical legislation either local or general, or both. Much tendency is shown by many of the public to look lightly upon the recent propagandas against the fly and the hook worm, both of which great dangers to man being often referred to in a spirit of derision and jocularity. We must be "Men from Missouri" and show the dear people that we are seeking to compass their safety, save them from ills and death. All this will require patience, but it will come as the public is by education made to recognize the sincerity of the medical profession in its efforts at disease prevention. Then will we be able to secure local city ordinances looking to the destruction of the typhoid fly and the hookworm by proper hygiene in and around the home. But a few years ago it was an unusual sight to meet an individual with the unsightly scars left by small pox. The public generally looked with dread on this loathsome disease. There are now many evidences that an inexplicable indifference has supplanted this dread. It has become in many sections, certainly in my part of the state, a very difficult matter to induce the county or municipal authorities to either isolate or vaccinate, and not only negroes, but even some whites go and come as they please with the pustules of variola full grown and evident to every one, and a pitted countenance is no uncommon sight. This is a deplorable situation and should be speedily remedied. I would suggest that every doctor in the state should urge the Representatives from his county and the Senator from his district to secure at the approaching State Legislative session the passage of a State law requiring every child of school age in the state to be properly vaccinated before it can again enter the public schools of the state, either white or colored. In this way small pox would soon be banished from the state for lack of materials to feed upon. If local, county and municipal authorities have come to think more of the relatively small cost of isolation and vaccination to control this disease than of the suffering, loss of time and deformities produced by it, let the State Legislature bring the desired end by the means suggested. In concluding this paper, permit me to briefly recapitulate by saying: Educate the public in matters of hygiene and disease prevention, secure a more real literary educational restriction in our medical college requirements, and thus raise to a still higher standard the medical man of Georgia.

